# Variation in plant functional groups indicates land degradation on the Tibetan Plateau

**DOI:** 10.1038/s41598-018-36028-5

**Published:** 2018-12-04

**Authors:** Jiufu Luo, Xuemin Liu, Jun Yang, Yuguo Liu, Jinxing Zhou

**Affiliations:** 1 Key Laboratory of State Forestry Administration on Soil and Water Conservation, School of Soil and Water Conservation, Beijing Forestry University, Haidian District, Beijing, 100083 China; 2 Jianshui Research Field Station, School of Soil and Water Conservation, Beijing Forestry University, Beijing, 100083 China; 3 Ministry of Education Key Laboratory for Earth System Modeling, Center for Earth System Science, Tsinghua University, Haidian District, Beijing, 100084 China; 4 Institute of Desertification Studies, Chinese Academy of Forestry, NO. 10 Huaishuju Road, Haidian District, Beijing, 100091 China

## Abstract

Plant functional groups (PFGs) have been increasingly introduced in land degradation (LD) studies; however, it is unclear whether PFGs can indicate LD. Here, we selected five different degraded lands (i.e., pristine and, lightly, moderately, seriously and extremely degraded) higher than 4650 m on the Tibetan Plateau. In addition, we investigated floristic metrics (i.e., composition, height, cover, biomass and abundance) and soil conditions (e.g., moisture, temperature and gravel ratio) by sampling 225 subplots. We found 75 vascular plants that consist of sedges (Cyperaceae), grasses (Gramineae), legumes, forbs, cushion plants and shrubs PFGs. LD dramatically deteriorated soil conditions, vegetation cover and productivity, however, improved species diversity. Moreover, cover and productivity showed a hump-shaped relationship with LD intensification in legumes, grasses and forbs and decreased mainly in sedges. Productivity increased considerably in cushion plants and shrubs on the extremely degraded land. Major characteristics of the LD process were the replacement of *Kobresia* spp. by *Carex* spp. in sedges; cushion plants significantly expanded, and shrubs appeared on the extremely degraded land. We, thus, confirm that the PFG variations are likely to indicate a LD process and demonstrate ways of using PFGs to assess LD status on the Tibetan Plateau.

## Introduction

Land degradation (LD) is a global concern because of its greatly negative influence^1–3^. Long-term, unsustainable practices on marginal lands have intensified LD visibly, especially for the overgrazing regime on grasslands on the Tibetan Plateau, which is the highest (mean elevation of 4000 m) and largest (2.0 × 10^6^ km^2^) plateau and is known as the Third Pole on the earth^4,5^. The degraded grasslands exceeded 5.0 × 10^5^ km^2^ with an annual degradation rate of 8%^6^, and the alpine cold swamp/meadow declined over 36% sharply in just 15 years after 1990^7^. Extreme LD has occurred over the past decades, reduced the biological or economical productive capacity and hindered regional development^3,8^. At the same time, the alpine meadow degradation has been a hot topic in ecological studies^9–11^. However, previous studies have focused on vegetation productivity or vegetated cover^12,13^, community species diversity^14,15^, or soil nutrients^14,16,17^ in degraded lands. Knowledge of the indicative plant functional group (PFG) in degraded alpine meadows is still rare^2^.

A PFG is considered to be a combination of species that possesses similar strategies to cope with similar environments^18^. Usually, plants in grassland communities are classified into sedges (Cyperaceae), grasses (Gramineae), forbs, vines, ferns or shrubs PFGs based on the plant growth form^19^. Evidence suggests that a PFG investigation is crucial for degraded grassland community structure or function research^9,20^, such as in a Siberian lowland tundra, where the shrubs (e.g., *Betula nana*), grasses (e.g., *Arctagrostis latifolia*), and sedges (e.g., *Eriophorum* spp.) cover ratio showed a close relationship with LD, resulting in thaw pond formation^21^. Yang *et al*. also found that not only the total number of vascular plants, but also the grasses or sedges cumulative density per unit area greatly decreased over the course of LD on the Tibetan Plateau^22^. Additionally, more detailed groups were established and studied due to special functions, such as nitrogen fixation of the legume PFG^2^. The legume PFGs promoted floral succession in degraded grasslands^2,23^, for example, *Medicago sativa* (legume) facilitated species recruitment and grassland productivity greatly^24^. However, Khan *et al*. argued that the facilitation of legumes was restricted by the dominant composition and habitat condition^25^. Moreover, among the extraordinary abundance of plant growth forms, cushion plants were noted early in alpine ecosystems, such as the Andes, Patagonia, Tierra del Fuego, New Zealand Alps, and in the Third Pole^26^. For over a century, cushion plants have fascinated many botanists and ecologists, such as *Silene acaulis* as a model species to explore cushion plant ecophysiology and population ecology^27,28^. However, cushion plants have rarely been separated from other PFGs and addressed as an individual PFG in previous studies^29^. Thus, some special PFGs have been ignored in previous studies, especially some unique PFGs in alpine ecosystems.

Here, we determine whether there is a consistent trend in all the well-founded PFGs in a certain alpine grassland in terms of their responses to the LD process. There has been no systematic, precise, or universal theory until now. In order to resolve these questions, we conducted a comprehensive investigation and PFG study during a LD process in the core area of the Tibetan Plateau. Moreover, we considered all the established PFGs based on the existing PFG division theory. With an ongoing effort to determine (1) the variations in PFG structure and aboveground productivity responses to a LD process filter and (2) whether such variations could indicate a LD process. Experimental sites were selected in an area undergoing a degradation process and the design followed a comprehensive ecological field experiment in the Tibetan Plateau alpine meadow region. A floristic composition investigation (identification, height, cover, abundance and aboveground biomass of vascular plants) was conducted, and the topsoil layer conditions (water content, temperature and gravel ratio) were measured.

## Results

### Floristic composition and soil condition variations during a LD process

Seventy-five vascular plant taxa distributed in 49 genera and 20 families of angiosperms were recorded in 225 subplots across the 5 communities (I = pristine land, II = lightly degraded land, III = moderately degraded land, IV = seriously degraded land and V = extremely degraded land) (Table S1). The γ-diversity from I to V was 23, 36, 34, 31 and 32, respectively. Similarly, the number of families and genera in I was less than that in the remnant communities. Furthermore, the mean α-diversity was the lowest in I (*P* < 0.05), increased from I to III and then decreased (Table 1). The floristic composition in sedges presented a qualitative change in that *Kobresia* spp. (e.g., *K. humilis*, *K. tibetica*, *and K. royleana*) were replaced by *Carex* spp. (e.g., *C. montis*-*everestii* and *C. moorcroftii*) from I to V. *Kobresia* spp. were the main dominant species in I and II, while *Carex* spp. (e.g., *C. montis*-*everestii*) and legumes (e.g., *Astragalus confertus*), grasses (e.g., *Stipa purpurea*), and forbs (e.g. *Leontopodium nanum*) were dominant in III, IV, and V. The sum of the importance values of *Kobresia* spp. decreased from 74.75 in I to 0 in V, while that of *Carex* spp. increased from 0 in I to 33.84 in V. *Androsace tangulashanensis* (cushion plant) was one of the top three dominant species in V (Table S1).

**Table 1:** Summary of the results of vascular plant diversity, vegetation cover and biomass, and soil conditions (top 10 cm depth) from recorded data; the different letters following the results indicate significant differences among communities (*P* < 0.05); DF means degree of freedom (between groups, within groups).

	I	II	III	IV	V	DF	*F*-value	*P*-value
Gravel % (SE)	0a	0a	18.45(1.40)b	29.80(3.50)bd	34.59(2.23)cd	4,20	68.538	<0.001
Moisture % (SE)	61.02(2.89)a	8.15(1.08)bc	6.67(0.42)b	5.06(0.27)bc	4.24(0.35)bc	4,20	935.938	<0.001
Temp. ( C) (SE)	9.21(0.27)a	10.48(0.26)b	13.10(0.34)c	13.42(0.32)c	15.46(0.38)d	4,20	20.360	<0.001
Slope (°)	<0.5°	<0.5°	<0.5°	<0.5°	<0.5°	—	—	—
Altitude (m)	4650	4656	4676	4680	4681	—	—	—
Vegetation cover %	89.49(1.58)a	74.73(1.93)b	50.73(2.08)c	30.33(1.64)d	20.47(1.21)e	4,20	311.213	<0.001
Total biomass (g·m^−2^)	122.70(8.54)a	62.13(8.41)b	35.60(2.28)c	17.06(2.13)d	17.77(2.01)d	4,20	52.703	<0.001
α-diversity (SE)	16.80(0.86)a	25.60(1.29)b	30.00(0.45)c	21.20(1.20)d	24.60(0.51)b	4,20	28.474	<0.001
γ-diversity	23	36	34	31	32	—	—	—
Families	10	15	13	13	16	—	—	—
Genera	18	30	26	25	23	—	—	—

**Figure 1:**
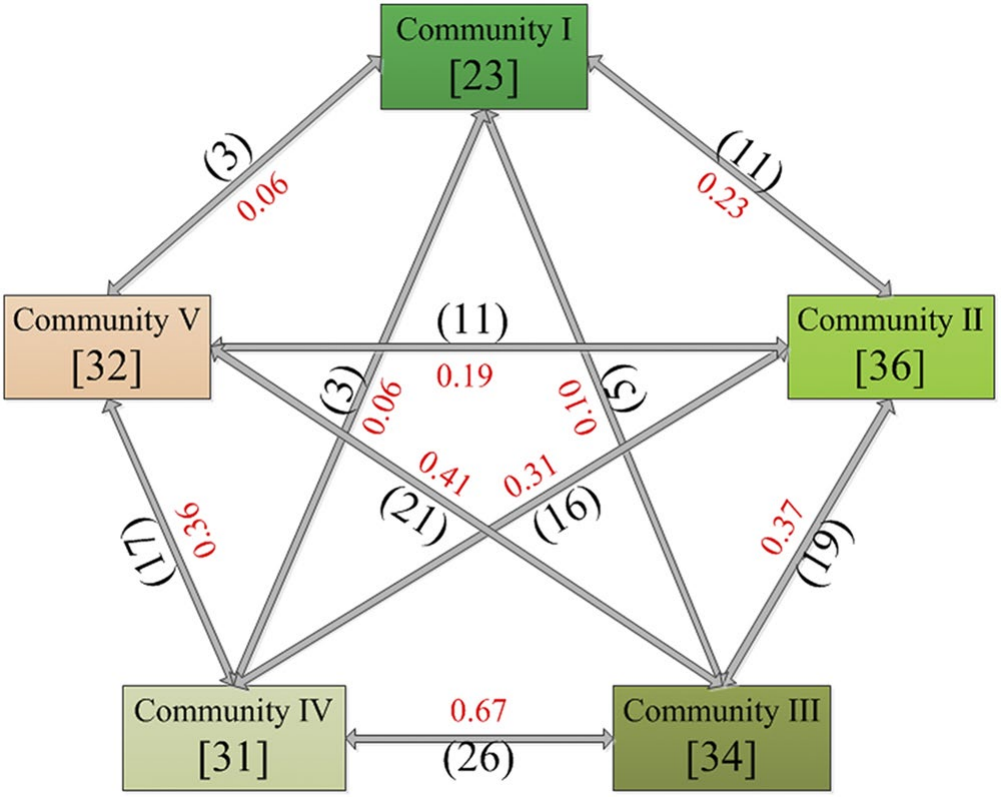
Vascular plant γ-diversity in each community and the Jaccard index among the five communities. The numerical values in the rectangles indicate the γ-diversity, the black numerical values in the parenthesis close to the arrows indicate common species number, and the red numerical values are the similarity index values.

The Jaccard index of total vascular plant composition in paired I–II, I–III, I–IV, and I–V decreased considerably (from 0.23 to 0.06), and the Jaccard index in paired II–III, II–IV, and II–V decreased from 0.37 to 0.19. In addition, II–IV (0.67) and III–V (0.41) presented higher similarity than the others, which indicated a community assembly transition during the LD process (Fig. 1).

The topsoil layer conditions presented notable variance. The gravel mass ratio and temperature increased greatly from I to V (*P* < 0.05), while the soil water content conversely declined markedly from I to V (*P* < 0.05) (Table 1).

### LD influences PFG structure

The sedge and grass γ-diversity obviously decreased in V in comparison to the other communities, whereas the legume and forb γ-diversity was lowest in I. There were three cushion plant taxa in V but only one in the remaining communities, and shrubs were solely observed in V (Table 2). The vascular plant similarity of each PFG among the five communities showed dramatic differences, and III, IV, and V had no common sedge or legume plants with I. In addition, the only cushion plant in I (*A. zambalensis*) was not present in the remaining communities. The paired communities of III, IV, and V had a relatively higher Jaccard index in each PFG (Tables 2, S1).

**Table 2:** The γ-diversity per PFG and Jaccard index values among the PFGs.

			PFGs					
			Sedges	Grasses	Cushion plants	Legumes	Forbs	Shrubs
γ-diversity of PFGs		I	4	4	1	2	12	0
		II	5	6	1	3	21	0
		III	3	6	1	7	17	0
		IV	3	4	1	6	17	0
		V	2	2	3	7	17	1
Jaccard Index	I	II	0.29	0.43	—	0.25	0.18	—
		III	—	0.43	—	—	0.07	—
		IV	—	0.33	—	—	0.04	—
		V	—	0.20	—	—	0.07	—
	II	III	0.33	1.00	1.00	0.25	0.27	—
		IV	0.33	0.67	1.00	0.13	0.27	—
		V	0.40	0.33	0.33	0.11	0.12	—
	III	IV	1.00	0.67	1.33	0.44	0.70	—
		V	0.67	0.33	0.33	0.75	0.40	—
	IV	V	0.67	0.20	0.33	0.63	0.31	—

**Figure 2:**
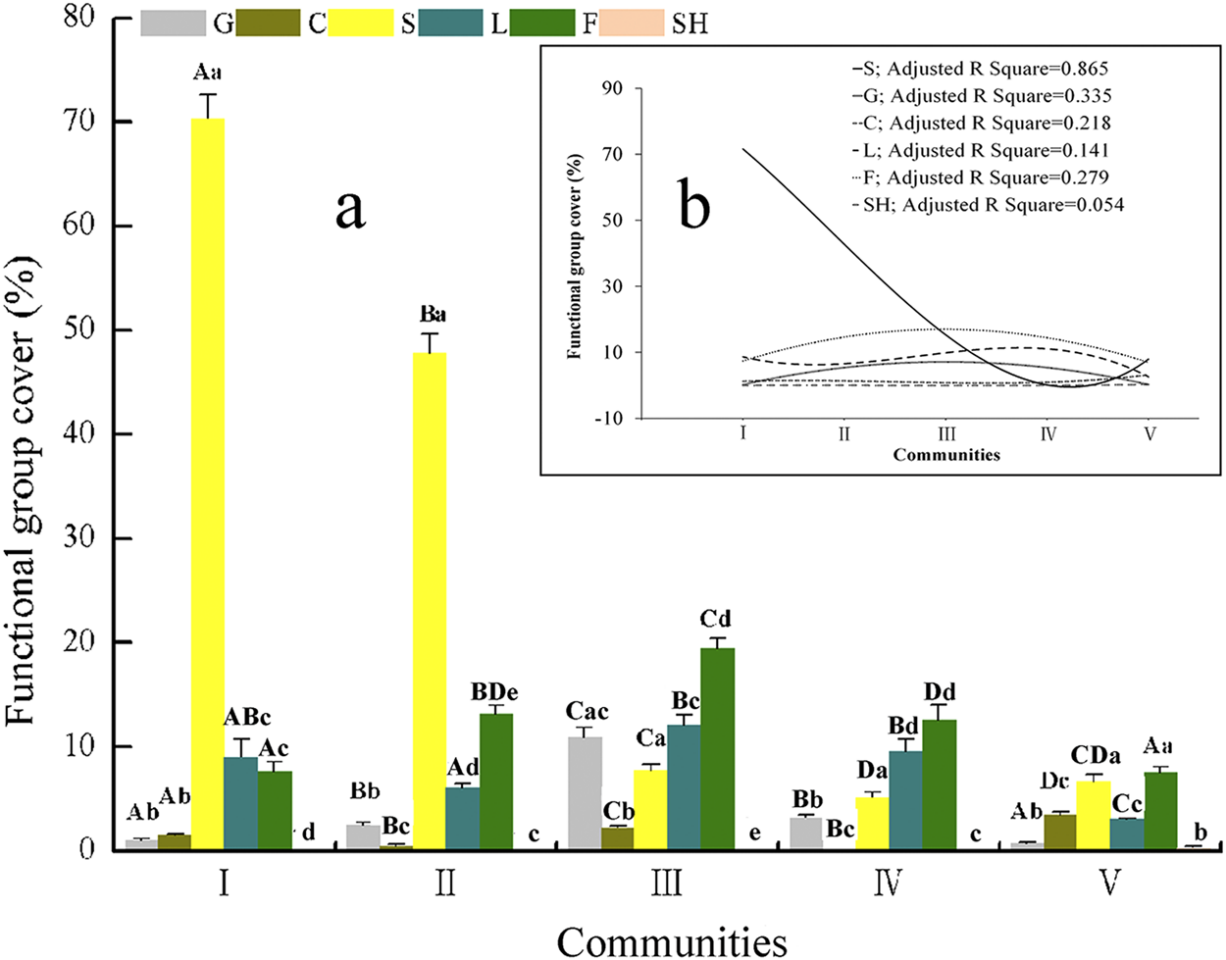
PFGs cover variations during the LD process. (**a**) The different capital letters indicate significant difference of a PFG among communities, and the different lowercase letters indicate significant differences in 6 PFGs in a certain community (*P* < 0.05) (the sample size n = 45 per community). (**b**) The best-fitting model that described PFG cover responses to the LD process.

Different response patterns of PFG cover in response to the LD gradient were observed (Fig. 2; Table S3). The cover of forbs, grasses and legumes markedly increased from I to III and then decreased (Fig. 2a), which indicated a hump-shaped trend (Fig. 2b; Table S3). Shrubs appeared solely in V. The cushion plant cover increased remarkably in V (15.44% cover ratio) and occupied a critical position in V (e.g., the importance value of *A. tangulashanensis* was as high as 5.12) (Fig. 2a; Tables S1, S2, S4). The results showed that all the response models were nonlinear. The sedge cover showed a cubic relationship (R^2^ = 0.865, *P* < 0.05) that significantly decreased from I to IV but weakly increased in V (Fig. 2; Table S3).

### LD influences PFG productivity

**Figure 3:**
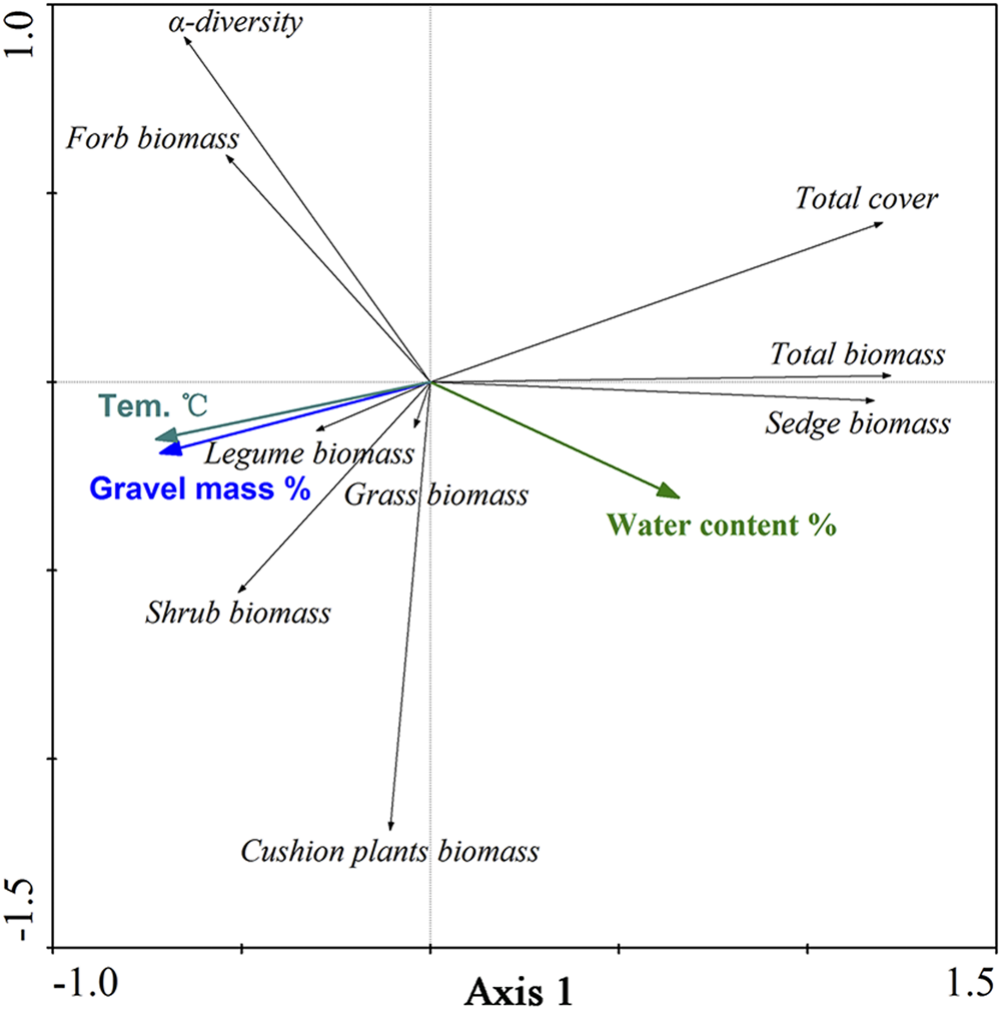
Redundancy analysis (RDA) for the relationships between topsoil conditions (gravel mass ratio, water content and temperature) and floristic metrics (α-diversity, PFG biomass, total community biomass and total vegetation cover). The figures were created with Canodraw for Windows 4.5.

The cumulative percentage variance in the relationship between plant community and environmental factors was 66.5% on axis 1 in the Redundancy Analysis (RDA) (Fig. 3). Axis 1 mainly reflected the LD process (r = −0.94 for topsoil temperature, r = 0.85 for topsoil water content, and r = −0.92 for topsoil gravel mass ratio). Similar to the total vegetation cover, the sedge and total community aboveground biomasses were positively correlated with topsoil moisture, whereas the forb, legume, grass, cushion plant and shrub aboveground biomasses were negatively correlated with topsoil moisture but positively correlated with topsoil temperature and gravel mass ratio.

**Figure 4:**
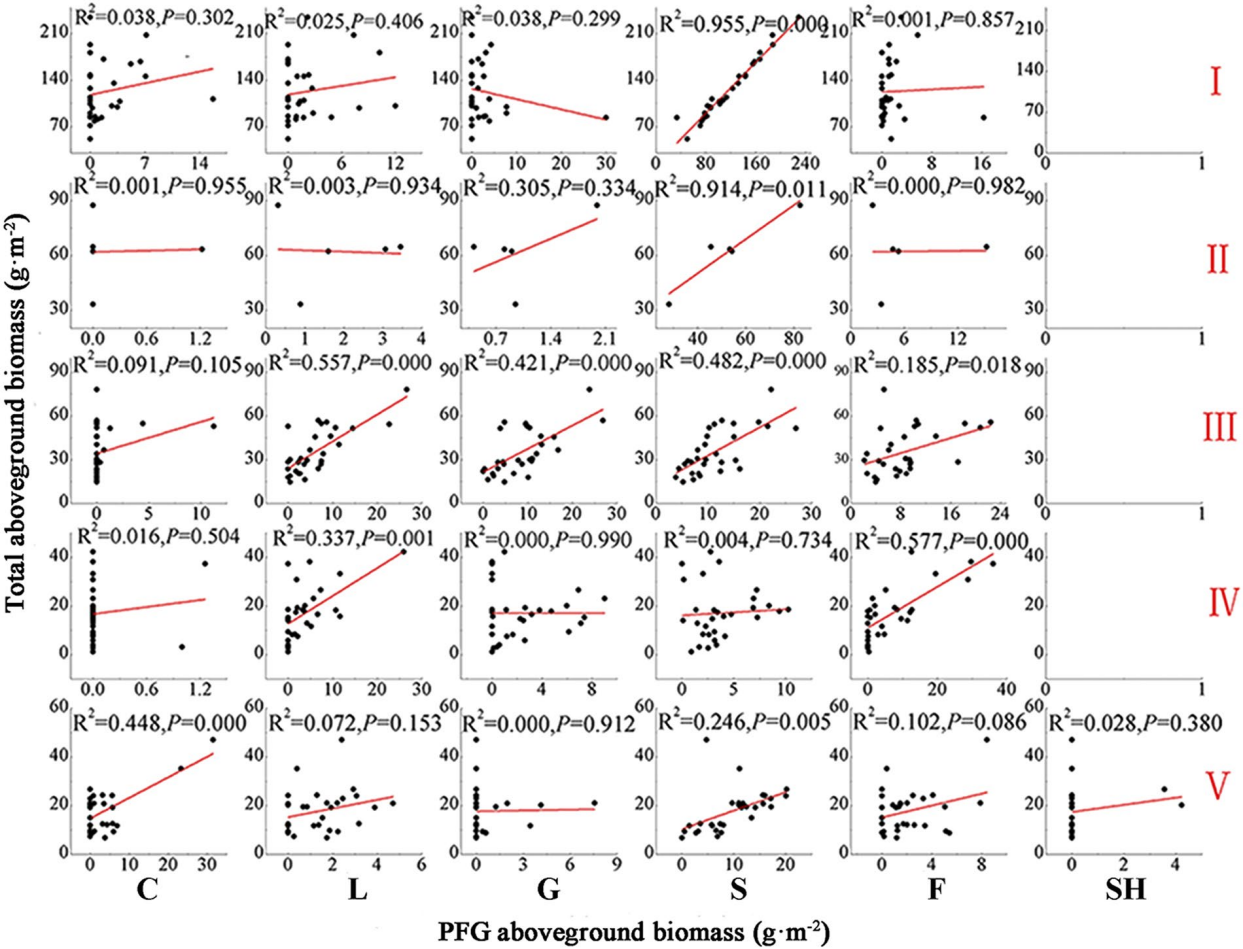
Relationships between total aboveground biomass and PFGs aboveground biomass during the LD process. R^2^ values indicate the strength of the relationship. The sample size n = 30, 5, 30, 30, 30, in I, II, III, IV, and V, respectively.

The general linear regression model showed substantially different contribution rates of each PFG to community productivity during the LD process. The productivity of I or II was based mainly on sedges (Fig. 4, *P* < 0.05; R^2^ = 0.955 in I and R^2^ = 0.914 in II), and legumes, grasses, sedges and forbs greatly contributed to the productivity of III (Fig. 4, *P* < 0.05; R^2^ = 0.557, 0.421, 0.482, and 0.185 for legumes, grasses, sedges and forbs, respectively). Moreover, the community productivity in V was mainly based on cushion plants (17.74% biomass ratio) and sedges (55.06% biomass ratio) (Fig. 4, *P* < 0.05; R^2^ = 0.448 and 0.246, respectively; Tables S2, S4).

## Discussion

Vast variations, including floristic composition, PFG structure and productivity, among all the PFGs during the LD process were observed in our study. There was no general response trend of the different PFGs to the LD process, as in previous studies. However, there were some differences that cannot be ignored in contrast to previous studies, such as the dominant palatable sedges or grasses declined significantly or disappeared or was replaced by an unpalatable forb (e.g., *Veratrum lobelianum*) during the LD process in the headwaters of the Yellow River, Caucasus Mountains or Siberian lowland tundra^21,30–32^. In our study, however, the grasses and legumes did not directly decrease with the intensification of LD but increased from pristine land to moderately degraded land; forbs did not replace sedges in the degraded lands, though the key species in the alpine meadow in our study region, *Kobresia* spp. (sedges), disappeared in the degraded lands gradually, and some other vascular plants, *Carex* spp. belonging to the sedges, expanded rapidly. This dispersal strategy may closely correlate with the evolutionary processes of local adaptation and niche separation of sedges^33^. *C. montis*-*everestii* and *C. moorcroftii* are guerrilla-type rhizomatous sedges with high cold and barren area tolerance, an exploitation resource strategy, and less habitat-dependent plasticity^34^. The developed guerrilla rhizome competes for belowground resources in degraded lands^35^, whereas *Kobresia* spp. in our study area are characterized by a phalanx growth form with a resource-acquisitive strategy^36^. This differentiation will help improve the accuracy of research on PFG ecological strategies in alpine degraded ecosystems and suggests that more attention should be given to the specific ecological strategies of PFGs along ecogeographical gradients^37^.

The α-diversity was higher in the degraded lands, especially in the land which presented higher grass and legume cover compared to the species loss in the previous studies^15,31^. It may be that the graminoids or legumes could benefit the vegetation or soil and establish higher species abundance as in the Greater Caucasus^2,38^. Furthermore, specific reasons may also account for these differences. The pristine land presented as densely and highly vegetated areas which visually thick mattic epipedon underground, which hinders species invasion, ecesis, growth, and reproduction through intra-and interspecific competition. Thus, it led to lower population diversity and lower floristic composition similarity than the degraded lands^10,39^. In addition, the dwarf height of dominant (i.e., *K. pygmaea*) and track patches provided niches for the invasions, such as the accidental species *Microula tibetica*, *Axyris prostrate*, and *Actinocarya tibetica* and increased the total species richness in lightly degraded land^10,38^. The high gravel cover property, therefore, provided more niches for the cold xerophytic/mesophytic grasses, legumes, or forbs, such as *Stipa purpurea*, *Roegneria thoroldiana*, *Oxytropis glacialis*, or *Cortiella caespitose*, and resulted in higher species diversity^40^. Thus, this variation in degraded lands, in contrast to pristine land, might demonstrate restoration opportunities in such poorly vegetated areas^2^.

Shrubs (*Myricaria prostrata*) only appeared in the extremely degraded land in our study area. Moreover, in contrast to the high-frequency studies of sedges, grasses, legumes, and forbs ecological strategies in previous studies, the prominence cover ratio (15.44%) and productivity ratio (17.74%) of cushion plants in extremely degraded land attracted our attention. We hypothesized that shrub appearance and the substantial expansion of cushion plants may indicate severe LD. These plants act as preservers of ecosystem function in the fragile and extremely degraded alpine region due to their efficient trapping of heat and water^26^. Hence, they were able to cope with the severely degraded land and serve as important ecosystem engineers in the alpine ecosystems^39,41,42^. Acting as nurse plant species, their expansion may support other plant growth within their tightly knit canopies and present a visible vegetated landscape in severely degraded land^43–45^. This species expansion may contribute to higher species diversity in extremely degraded land as they are a long-lived PFG^46^. More specifically, this expansion may also demonstrate the indicator function of cushion plants and shrubs for severe LD on the alpine plateau.

Finally, we found that PFG variations may indicate a LD process on the alpine grasslands of the Tibetan Plateau. Our results showed that LD increased vascular plant diversity but deteriorated community productivity, cover and soil conditions. The ongoing intensified LD was accompanied by sedges assembly replacement of *Kobresia* spp. by *Carex* spp. and sedges cover and productivity declined as a whole. The cover and productivity of forbs, grasses and legumes showed a hump-shaped trend with LD intensification. Shrub and cushion plants expanded greatly in the extremely degraded land. This study may provide research recommendations and methods for exploring LD by a PFG analysis.

## Materials and Methods

### Study area

**Figure 5:**
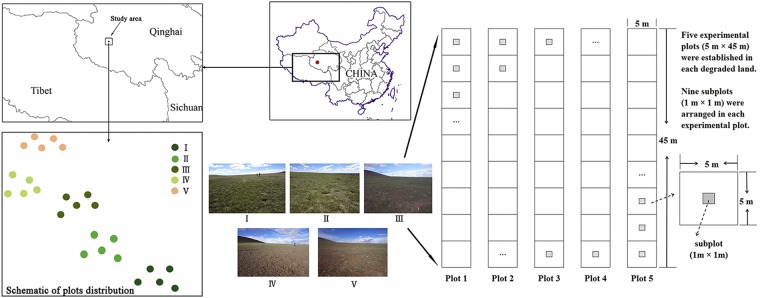
Schematic drawing of the field experimental designs in the study area. The map was created with ArcGIS v10.2 (http://www.esri.com/arcgis/about-arcgis).

The field experiment was performed at the Tibetan Plateau Research Base of the State Key Laboratory of Permafrost Engineering, Chinese Academy of Sciences (34°49′16.5″–34°49′44.9″N, 92°55′14.6″–92°56′35.4″E; 4650 m a.s.l) (Fig. 5). The research base is located in the Three River source region, which has a typical plateau alpine climate with a mean annual temperature and precipitation of −3.8 °C and 290 mm, respectively. Precipitation mainly falls in the vegetation growing season (May to October)^47^. In general, there is a frozen period every year (September to the next April)^48^. Human-induced degradation, such as the overgrazing regime, was the primary cause of degradation in the grassland, and there were approximately 15.41 million sheep units in the Three River source region (approximately 0.36 million km^2^) during the year 2003–2012^49,50^. The grassland is grazed by yaks, *Bos grunniens*, and sheep, *Ovis aries*, in the summer season^48^. Vegetation damage caused by yaks or sheep grazing, and Tibetan herdsmen activities can be observed in this area. The vegetation cover ranges from less than 10% to more than 90%, presenting different types of degraded landscapes^38,51^. The study area is dominated by a typical alpine swamp meadow, the climatic climax in this region, with cold-adapted mesophytic perennial herbs, such as *Kobresia tibetica*, *K. humilis* and *K. pygmaea*. The degraded grassland landscape (alpine cold steppe or alpine desert) also occupies a large area. The alpine cold steppe mainly consists of xeric grasses, such as *Stipa purpurea* and *Littledalea racemosa*. The alpine desert is dominated by xerophyte or drought-enduring plants, such as *Leontopodium nanum* and *Saussurea arenaria*^52^.

### Experimental design

A comprehensive field experiment was designed based on a field investigation in our study area. Five different degraded alpine communities, I = pristine land, II = lightly degraded land, III = moderately degraded land, IV = seriously degraded land and V = extremely degraded land (thereafter, I, II, III, IV, and V), were characterized by combining the preliminary fieldwork investigation with the previous studies in this area^31,51,53^ (Fig. 5). Then, we investigated the floristic composition and topsoil condition. I is alpine swamp meadow that has the highest vegetation cover in the study area; II is an alpine meadow with relatively high vegetation cover but lower than I visually; III is an alpine steppe landscape that has a relatively high soil bareness ratio; in addition, IV and V are typical alpine desert landscapes, of which V has the highest bareness degree (see the colour photos shown in Fig. 5).

Five experimental plots (5 m × 45 m) were established in each community with a regular pattern. In addition, nine subplots (1 m × 1 m) were arranged in each plot, so that there were 45 subplots in each community^9,31,38^. The resulting data analysed in this study consisted of 225 subplots distributed across 25 plots (Fig. 5).

### Floristic composition investigation and PFG classification

The investigation was conducted during the growing season. Within each subplot, all vascular plant taxa were recorded. Botanical nomenclature followed “Flora of China” (http://foc.eflora.cn/). The number of vascular plants was counted in each subplot, and the number of monocotyledons was counted by the ramets. Average plant height was measured by a ruler separately for each species in all subplots. The cover of vegetation and plant populations in each subplot was estimated by visual inspection based on the previous study^2^. Thirty subplots in I, III, IV, and V and five in II (for which the community was relatively homogeneous) were selected randomly to harvest the aboveground biomass by cutting the plants close to the ground after the composition investigation. All harvested plants were dried at 65 °C until they reached a constant weight^47,48^.

Six PFGs were considered: sedges (namely, Cyperaceae plants), grasses (namely, Gramineae plants), legumes (namely, Leguminosae plants), cushion plants (we selected spongy cushion plants that are typical cushion plants to research)^26^, forbs (the rest of herbaceous plants), and shrubs. We regarded grasses and sedges as separate groups rather than a general graminoid group because of the predominance of sedges in the alpine meadow in this study region.

### Soil condition data collection

At the same time as the floristic composition investigation, we monitored the topsoil (depth of 10 cm) temperature using iButton Hygrochron temperature logger (DS1923) at all 25 plots. Nine topsoil samples were sampled randomly by a hand shovel to obtain the gravel accurately in each plot and pooled as a soil mixture sample (approximately 500 g). Thus, a total of 25 soil mixture samples were collected from 225 samples. Approximately 50 g (2 mm) soil was sieved quickly from it to measure the topsoil water content using the gravimetric method by drying samples at 105 °C^8^. The remnant soil was air-dried for seven days and sieved to obtain gravel (>2 mm). All plant litters or roots were removed and weighed to calculate the gravel mass ratio^38^. To eliminate weather influence (e.g., rainfall and temperature) on soil moisture, we collected soil samples in five communities as soon as possible on a sunny day.

### Statistical analysis

Vascular plant diversity was presented based on the α-diversity (defined as species richness of a single plot) and γ-diversity (defined as the total species richness of 5 plots in each community)^54–56^.

The floristic composition data from each community were simplified to calculate the importance value (*IV*) of each vascular plant using the following equation (Equation 1):

1 $$IV=\frac{RC+RH+RD}{3}$$

where RC, RH, and RD represent the relative cover, relative height and relative density of the species, respectively.

The floristic composition similarity between communities and PFGs was tested using the Jaccard index^57^ (Equation 2):

2 $$Jaccard\,index=\frac{j}{a+b-j}$$

where *j* is the number of common vascular plants in paired communities, and *a* and *b* are the number of vascular plants in paired communities.

Because different plant communities and soil conditions were observed in the different degraded lands, analysis of variance (ANOVA) with least significant difference (LSD) (for equal variances) or Dunnett’s T3 (for unequal variances) based on the *F*-statistic was used to test the significance of species diversity, vegetation cover, total biomass, PFGs cover and biomass, and topsoil conditions during the LD process (*P* < 0.05, IBM SPSS Statistics 20.0 for Windows)^2,58^. A redundancy analysis (RDA) was used to assess the effects of topsoil conditions on plant community characteristics as described previously based on the data in each plot (Canoco for Windows 4.5)^2^. The plant data were log transformed, centred and standardized when they were subjected to the RDA^59^.

General linear regression analysis was performed to test the strength of the relationship between the total aboveground biomass and the PFG aboveground biomass in each community. Models that described PFG cover responses to degradation status were calculated, and the best-fitting model was selected based on the coefficient of determination R^2^ (ranging from 0 to 1)^60^. Because some of the PFG variables contained zero values, log transformation could not be applied. Therefore, we tested the linear, logarithmic, inverse, quadratic, and cubic models. The figures were created with OriginPro 2016 (OriginLab Corporation, Northampton, MA, US) and combined by Adobe Photoshop CS6 v6.0.335.0.

## Electronic supplementary material

Supplementary Information

## Data Availability

The datasets generated during and/or analysed during the current study are not publicly available due to an ongoing further study but can be requested from the corresponding author.
