# Supplementary material for: Variation in plant functional groups indicates land degradation on the Tibetan Plateau
Source: Sci Rep. 2018 Dec 4;8:17606. doi: 10.1038/s41598-018-36028-5 (PMC6279773; doi:10.1038/s41598-018-36028-5)
Supplement: Supplementary file 1 — Supplementary Information [file 41598_2018_36028_MOESM1_ESM.pdf]

Title page

Full title: Variation in plant functional groups indicates land degradation on the Tibetan Plateau

Authors and addresses

Jiufu Luo[1,2]

[1]Key Laboratory of State Forestry Administration on Soil and Water Conservation, School of Soil and Water Conservation, Beijing Forestry University, Haidian District, Beijing 100083, China.

[2]Jianshui Research Field Station, School of Soil and Water Conservation, Beijing Forestry University, Beijing 100083, China.

Xuemin Liu[1,2]

[1]Key Laboratory of State Forestry Administration on Soil and Water Conservation, School of Soil and Water Conservation, Beijing Forestry University, Haidian District, Beijing 100083, China.

[2]Jianshui Research Field Station, School of Soil and Water Conservation, Beijing Forestry University, Beijing 100083, China.

Jun Yang[3]

[3]Ministry of Education Key Laboratory for Earth System Modeling, Center for Earth System Science, Tsinghua University, Haidian District, Beijing 100084, China

Yuguo Liu[4]

[4]Institute of Desertification Studies, Chinese Academy of Forestry, NO.10 Huaishuju Road, Haidian District, Beijing, 100091, China.

***Jinxing Zhou**[1,2]

[1]Key Laboratory of State Forestry Administration on Soil and Water Conservation, School of Soil and Water Conservation, Beijing Forestry University, Haidian District, Beijing 100083, China.

[2]Jianshui Research Field Station, School of Soil and Water Conservation, Beijing Forestry University, Beijing 100083, China.

*Corresponding author

J. Z., email: zjx001@bjfu.edu.cn; Tel: 86-10-6233-8561

Table S1. The floristic composition and the species importance values.

	**Vascular plant serial number (importance value)**
I	**37**(30.41), **36**(15.63), **32**(13.27), **52**(6.72), **9**(4.44), **55**(3.50), **58**(2.91), **61**(2.24), **4**(2.00), **41**(0.71), **62**(0.60), **40**(0.50), **50**(0.44), **28**(0.33), **38**(0.27), **63**(0.14), **7**(0.12), **26**(0.10), **74**(0.10), **30**(0.06), **60**(0.06), **15**(0.03)
II	**34**(46.85), **18**(9.07), **41**(6.79), **52**(4.11), **33**(4.06), **66**(3.48), **40**(3.48), **32**(3.38), **57**(3.31), **60**(2.31), **55**(2.03), **70**(1.78), **24**(1.38), **74**(1.38), **46**(0.83), **65**(0.74), **75**(0.71), **8**(0.52), **39**(0.47), **56**(0.46), **38**(0.46), **72**(0.43), **31**(0.41), **71**(0.32), **29**(0.29), **54**(0.16), **17**(0.14), **16**(0.13), **25**(0.12), **1**(0.11), **11**(0.09), **28**(0.05), **44**(0.04), **14**(0.03), **7**(0.03), **43**(0.03)
III	**17**(12.62), **40**(10.82), **72**(10.43), **10**(8.08), **66**(5.64), **18**(5.38), **65**(5.06), **2**(3.77), **25**(3.47), **38**(3.39), **35**(2.63), **29**(2.46), **8**(2.15), **47**(1.94), **59**(1.90), **54**(1.70), **55**(1.57), **27**(1.52), **20**(1.38), **16**(1.16), **49**(0.82), **12**(0.55), **11**(0.47), **53**(0.45), **31**(0.43), **68**(0.40), **3**(0.31), **19**(0.29), **46**(0.18), **51**(0.16), **73**(0.09), **60**(0.03)
IV	**40**(19.64), **17**(16.76), **10**(12.64), **72**(11.15), **66**(7.37), **65**(5.82), **18**(5.34), **35**(5.19), **20**(2.88), **16**(2.35), **24**(1.90), **54**(1.59), **31**(1.04), **41**(0.88), **43**(0.78), **11**(0.74), **68**(0.68), **29**(0.51), **2**(0.47), **27**(0.37), **59**(0.33), **47**(0.32), **12**(0.27), **13**(0.26), **21**(0.25), **70**(0.15), **19**(0.10), **49**(0.07), **38**(0.06), **73**(0.06), **8**(0.08)
V	**17**(32.40), **40**(18.71), **4**(5.12), **48**(4.81), **2**(4.20), **47**(3.83), **25**(3.43), **24**(3.02), **41**(2.76), **68**(2.51), **64**(2.36), **8**(2.10), **3**(1.98), **51**(1.78), **10**(1.49), **18**(1.43), **12**(1.27), **54**(1.24), **23**(1.18), **20**(0.90), **22**(0.87), **45**(0.78), **53**(0.58), **69**(0.44), **6**(0.15), **9**(0.16), **27**(0.10), **42**(0.10), **49**(0.10), **67**(0.07), **66**(0.07), **11**(0.07)

Note: 1. *Actinocarya tibetica* Benth.; **2.** *Ajania tenuifolia* (Jacq.) Tzvel.; **3.** *Allium carolinianum*; **4.** *Androsace tangulashanensis* Y. C. Yang et R. F. Huang; **5.** *A. zambalensis* (Petitm.) Hand.-Mazz.; **6.** *Anemone imbricata* Maxim.; **7.** *Aphragmus oxycarpus*; **8.** *Arenaria bryophylla* Fernald; **9.** *Aster flaccidus* Bge.; **10.** *Astragalus confertus* Benth. ex Bunge; **11.** *A. hendersonii* Baker; **12.** *A. mattam* Tsai et Yu; **13.** *A. pulvinatus* P.C. Li et Ni, Acta Phytotax.Sin.; **14.** *Axyris prostrata* L.; **15.** *Braya rosea*; **16.** *Callianthemum pimpinelloides* (D. Don) Hook. f.et Thoms.; **17.** *Carex montis-everestii* Kükenth.; **18.** *C. moorcroftii* Falc. ex Boott; **19.** *Comastoma falcatum* (Turcz. ex Kar. et Kir.) Toyokuni; **20.** *Cortiella caespitosa* Shan et Sheh; **21.** *Corydalis fimbripetala* Ludlow; **22.**

Cremanthodium decaisnei; **23.** *Delphinium tangkulaense* W. T. Wang; **24.** *Draba altaica*; **25.** *Festuca brachyphylla* Schult.; **26.** F. ovina L.; **27.** *Gentiana crenulatotruncata* (Marq.) T. N. Ho; **28.** *G. leucomelaena* Maxim.; **29.** *Gentianella pygmaea* (Regel et Schmalh.) H. Smith; **30.** *Halerpestes filisecta* L. Liou; **31.** *Iris potaninii*; **32.** *Kobresia humilis* (C. A. Mey. ex Trautv.) Sergiev; **33.** *K. macrantha* Bocklr.; **34.** *K. pygmaea* C. B. Clarke; **35.** *K. robusta* Maxim.; **36.** *K. royleana* (Nees) Bocklr.; **37.** *K. tibetica* Maxim.; **38.** *Koeleria cristata* (L.) Pers.; **39.** *Lagotis brachystachya* Maxim.; **40.** *Leontopodium nanum* (Hook. f. et Thoms.) Hand.-Mazz.; **41.** *Littledalea racemosa* Keng; **42.** *Meconopsis horridula* Hook. f. et Thoms.; **43.** *Microula tibetica* Benth.; **44.** *M. younghusbandii* Duthie; **45.** *Myricaria prostrata* Hook. f. et Thoms. ex Benth.; **46.** *Oxytropis densa* Benth. ex Bunge; **47.** *O. falcata* Bunge; **48.** *O. glacialis* Benth. ex Bunge; **49.** *O. humifusa* Kar. et Kir.; **50.** *O. ochrocephala* Bunge; **51.** *O. platysema* Schrenk; **52.** *O. pusilla* Bunge; **53.** *Pedicularis alaschanica* Maxim.; **54.** *Pleurospermum hedinii* Diels; **55.** *Poa sinoglauca* Ohwi; **56.** *Polygonum sibiricum* Laxm.; **57.** *P. viviparum* L.; **58.** *P. viviparum* L. var. *angustum* A. J. Li; **59.** *Potentilla bifurca* L; **60.** *P. saundersiana* Royle; **61.** *Primula tibetica* Watt; **62.** *Ranunculus longicaulis* C. A. Mey. var. *nephelogenes* (Edgew.) L. Liu; **63.** *R. tanguticus* (Maxim.) Ovcz.; **64.** *Rhodiola quadrifida* (Pall.) Fisch. et. Mey.; **65.** *Roegneria thoroldiana* (Oliv.) Keng; **66.** *Saussurea arenaria*; **67.** *S. graminea*; **68.** *S. apus*; **69.** *S. subulata*; **70.** *Sibbaldia adpressa* Bge.; **71.** *Stellaria cherleriae* (Fisch. ex Ser.) Williams; **72.** *Stipa purpurea* Griseb.; **73.** *Taraxacum tibetanum* Hand.-Mazz.; **74.** *Thalictrum alpinum* L.; **75.** *Veronica biloba* L.

Table S2. The cover and aboveground biomass ration per plant functional group (PFG). Different letters indicate significant differences, the difference capital letter means the significant difference of each PFG during the land degradation (LD) process and the difference lowercase means the significant PFGs difference in a certain LD gradient (*P*<0.05).

		**Sedges**	**Grasses**	**Cushion plants**	**Legumes**	**Forbs**	**Shrubs**
PFGs aboveground biomass ratio (SE) (%)	I	92.55(1.99)Aa	2.65(1.23)Abc	1.57(0.51)Abc	1.78(0.51)Ab	1.45(0.66)Abc	0c
	II	84.18(3.91)Aa	1.70(0.39)Ab	0.39(0.39)Ab	3.16(0.89)ABb	10.57(3.48)ACb	0b
	III	33.39(2.32)Ba	24.14(2.59)Bac	1.16(0.75)Ab	15.17(2.04)Cc	26.14(2.31)BDa	0b
	IV	32.56(4.59)Ba	16.68(3.38)Ba	1.19(1.08)Ab	18.50(3.79)Ca	31.08(5.92)CDa	0b
	V	55.06(4.76)Ca	3.97(1.64)Abc	17.74(4.35)Bb	8.51(1.56)BCb	13.59(2.82)Cb	1.14(0.81)c
PFGs cover ratio (SE) (%)	I	78.87(1.55)Aa	1.24(0.19)Ab	1.70(0.12)Ab	9.54(1.63)Ac	8.65(0.81)Ac	0d
	II	68.90(1.26)Ba	3.62(0.34)Bb	0.56(0.19)Bc	7.39(0.60)Ad	19.54(1.05)Be	0c
	III	15.11(1.08)Ca	20.19(1.26)Cb	4.35(0.41)Cc	22.73(1.42)Bb	37.63(1.46)Cd	0e
	IV	18.60(1.80)Ca	12.05(1.23)Da	0.10(0.10)Bb	29.99(2.89)Bc	39.36(2.89)Cc	0b
	V	29.40(2.27)Da	3.62(0.56)Bb	15.44(1.29)Dc	15.27(1.06)Cc	34.78(2.04)Ca	1.49(0.75)b

Table S3. Summary of R^2 values for the models used to identify the threshold of the responses of the different PFG cover values during the LD process.

**PFGs**	**Linear**		**Logarithm**		**Inverse**		**Quadratic**		**Cubic**	
	$\mathbf{R^2}$	***P*-value**	$\mathbf{R^2}$	***P*-value**	$\mathbf{R^2}$	***P*-value**	$\mathbf{R^2}$	***P*-value**	$\mathbf{R^2}$	***P*-value**
Sedges	0.724	**<0.001**	0.811	**<0.001**	0.796	**<0.001**	0.855	**<0.001**	0.866	**<0.001**
Grasses	0.000	**0.970**	0.015	**0.063**	0.046	**0.001**	0.341	**<0.001**	0.343	**<0.001**
Cushion plants	0.073	**<0.001**	0.039	**0.003**	0.016	**0.062**	0.181	**<0.001**	0.228	**<0.001**
Legumes	0.020	**0.036**	0.010	**0.128**	0.006	**0.234**	0.074	**<0.001**	0.153	**<0.001**
Forbs	0.000	**0.775**	0.010	**0.139**	0.038	**0.003**	0.285	**<0.001**	0.285	**<0.001**
Shrubs	0.034	**0.006**	0.022	**0.025**	0.013	**0.085**	0.058	**0.001**	0.067	**0.002**

Table S4. A certain PFG cover, cover ratio and aboveground biomass ratio variances during the LD process and the PFGs cover, cover ratio and aboveground biomass ratio variances in a certain degraded land based on the ANOVA. Shown are the degree of freedom (DF, between groups, within groups) and the *F*-values with significance levels: [a] *P*-value >0.05, ** *P*-value <0.01, and *** *P*-value <0.001.

Factors		PFGs cover		PFGs cover ratio		PFGs aboveground biomass ratio	
		DF	*F*-value	DF	*F*-value	DF	*F*-value
PFGs	Sedges	4,220	465.003***	4,220	322.772***	4,120	48.916***
	Grasses	4,220	75.358***	4,220	87.540***	4,120	15.660***
	Cushion plants	4,220	46.820***	4,220	107.228***	4,120	10.203***
	Legumes	4,220	12.609***	4,220	30.222***	4,120	8.504***
	Forbs	4,220	26.629***	4,220	54.435***	4,120	11.395***
	Shrubs	4,220	4.006***	4,220	3.971**	4,120	1.549[a]
Communities	I	5.264	522.943***	5.264	982.282***	5,174	121.068***
	II	5.264	454.032***	5.264	1326.105***	5,24	233.299***
	III	5.264	97.676***	5.264	155.924***	5,174	51.202***
	IV	5.264	43.028***	5.264	70.918***	5,174	14.188***
	V	5.264	52.233***	5.264	82.667***	5,174	42.397***
